# Internalization of benzylisoquinoline alkaloids by resting and activated bone marrow-derived mast cells utilizes energy-dependent mechanisms

**DOI:** 10.1007/s00011-021-01526-2

**Published:** 2022-01-25

**Authors:** Syed Benazir Alam, Marianna Kulka

**Affiliations:** 1 Nanotechnology Research Centre, National Research Council Canada, 11421 Saskatchewan Dr NW, Edmonton, AB T6G 2M9 Canada; 2 Medical Microbiology and Immunology, University of Alberta, Edmonton, AB Canada

**Keywords:** Berberine, Lipoplexes, Endocytosis, Mast cells, Interleukin-3

## Abstract

**Objective and design:**

Drug delivery to inflammatory cells is dependent upon poorly understood, complex endocytic processes. Berberine (BBR), a benzylisoquinoline alkaloid, binds to heparin and targets glycosaminoglycan-rich granules in mast cells (MC), but the mechanism of BBR internalization is unknown.

**Methods:**

BMMC were treated with various concentrations of BBR for different amounts of time and BBR internalization was assessed by flow cytometry and fluorescence microscopy. BMMC were pretreated with endocytic inhibitors or a growth factor (IL-3) prior to BBR exposure to access mechanisms of its internalization.

**Results:**

After 24 h, 48 ± 0.8% of BMMC internalized BBR and this process was dependent upon temperature and the presence of glucose in the medium. Methanol fixation reduced BBR internalization, suggesting the involvement of an energy-dependent active transport mechanism. To determine mode of internalization, BBR was encapsulated into Lipofectamine TM lipoplexes since these are known to circumvent classical endocytic pathways. Incorporating BBR into lipoplexes decreased BBR internalization by 26% and 10% (10 μg/ml and 100 μg/ml Lipo-BBR respectively) by BMMC. BBR endocytosis was significantly reduced by Latrunculin B (88%), Cytochalasin B (87%), Chloroquine (86.5%) and 3-methyladenine (91%), indicating that actin polymerization, lysosomal pH and lysosomal self-degradation via the autophagy pathway was involved. In contrast, IL-3 treatment significantly enhanced BBR endocytosis (54% by 40 ng/ml IL-3) suggesting that IL-3 signaling pathways play a role in internalization.

**Conclusions:**

Our data suggests that internalization of BBR by resting and IL-3-activated BMMC utilizes an energy-dependent pathway that is dependent upon glucose metabolism and temperature. Furthermore, this process requires actin polymerization and lysosomal trafficking. These data suggest internalization of benzylisoquinoline compounds is an active and complex process.

**Supplementary Information:**

The online version contains supplementary material available at 10.1007/s00011-021-01526-2.

## Introduction

Mast cells are large granular immune cells that orchestrate homeostatic processes during inflammation, fibrosis, and infection [6]. Mast cells initiate and modulate key physiologic responses such as inflammation, tissue remodelling and immune responses to infection [7]. Within their densely packed intracellular granules, mast cells store several immunomodulatory mediators including tryptase, histamine, cytokines and chemokines within a proteoglycan matrix. When activated, mast cells release these mediators in a highly controlled process of exocytosis [1]. Like all cells, mast cells also internalize molecules from their microenvironment but the process of mast cell endocytosis, pinocytosis, or phagocytosis is poorly understood. Receptor-mediated endocytosis of the high affinity mast cell IgE receptor, FcεRI, is certainly an important process for its activation [2] and is dependent upon membrane gangliosides such as GD1b [3]. It has been postulated that mast cells internalize proteins and process them for antigen presentation via the major histocompatibility complex class I and II [4–6], although this process may not occur in all mast cell phenotypes. The process of mast cell internalization of small molecules, particularly inhibitors or therapeutic drugs, is even less clear. Since drug delivery applications rely heavily on these internalization processes, it is important to better understand mast cell internalization of such small molecules.

Berberine (BBR) is a quaternary ammonium salt from the protoberberine group of benzylisoquinoline alkaloids and when activated with ultraviolet light, BBR emits a strong yellow fluorescence [7–9]. Since BBR ions bind highly charged moieties such as heparin, BBR sulphate has been used as a histological stain of mast cell granules [10] and was one of the first methods used to analyze mast cells by flow cytometry [11]. BBR inhibits FcεRI-mediated signaling and mitogen-activated protein kinase (MAPK) phosphorylation in RBL-2H3 rat basophilic leukemia cells, often used as a model of mast cells [12]. Unlike large proteins, internalization of BBR is unlikely to occur via receptor-mediated endocytosis and may rely on energy-independent diffusion or unknown pinocytotic pathways dependent on actin polymerization.

In this study, we characterized the mechanism of BBR internalization by terminally differentiated bone marrow-derived mouse mast cells (BMMC). Our results show that internalization of BBR is significantly reduced when cells are fixed with methanol, suggestive of energy-dependent active transport of BBR across the cell membrane. Counterintuitively, protection of BBR within lipoplexes, conventionally considered to be a favourable method of drug delivery, inhibits BBR internalization into mast cells. Using a variety of inhibitors of the receptor mediated endocytosis pathway, we further show that BBR is internalized via autophagy-associated endocytic pathway. Activation of BMMC by interleukin 3 (IL-3), a differentiation factor for mouse mast cells, further potentiated BBR internalization.

## Materials and methods

### BMMC culture

Mice were euthanized by CO_2_ asphyxiation following isoflurane anesthesia. The tibia and femur were isolated and whole bone marrow was harvested. All animal studies were conducted in accordance with the Canadian Council on Animal Care Guidelines and Policies (https://ccac.ca/en/about-the-ccac/) with approval from the Health Science Animal Care and Use Committee for the University of Alberta. Bone marrow was aspirated using a 19 gauge needle and the cells were cultured in RPMI media (Fisher, Hampton, New Hampshire, USA) supplemented with 4 mM L-glutamine (Fischer), 50 μM BME (Sigma-Aldrich, Oakville, Ontario, Canada), 1 mM sodium pyruvate (Fischer), 100 U/ml penicillin/100 μg/ml streptomycin (Fischer), 0.1 mM nonessential amino acids (Fischer), 25 mM HEPES (Fisher), 10% FBS (Gibco, Burlington, Ontario, Canada) and 30 ng/ml mouse recombinant interleukin (IL)-3 (Peprotech, Rocky Hill, New Jersey, USA), pH-7.4–7.6, in a humidified atmosphere of 5% CO_2_ in air at 37 °C. This media will be referred to as “supplemented RPMI”. The cell suspensions were maintained at a density of 10^5^ cells/ml for 4 weeks when the cells were tested for FcεRI and c-Kit expression by flow cytometry to confirm maturation. After 4 weeks, 99% of cells were double positive for c-Kit and FcεRI. BMMC were used between 4 and 8 weeks of age.

### Treatment of BMMC with berberine, Lipofectamine 2000, methanol and inhibitors

BMMC (1 × 10^6^ cells/ml) were seeded into round-bottomed 96 well plates in supplemented RPMI media and treated with berberine chloride (BBR, Sigma Aldrich, B3251) dissolved in phosphate buffered saline (PBS) to a final effective concentration of 0, 0.01, 0.1, 1, 10 and 100 µg/ml. The BMMC were treated with BBR at 37 °C for 24 h. For the viability assay, BMMC were stained with trypan blue followed by counting and calculation of % viability and No. of live cells/ml.

BBR was complexed with Lipofectamine 2000 transfection reagent (Invitrogen, 11,668,027) according to the manufacturer’s instructions. Briefly, 500 µl of a 1 mg/ml BBR solution was mixed either with 15 µl of PBS or 15 µl of the Lipofectamine 2000 transfection reagent (equivalent of 15 µg). The solutions were mixed thoroughly by vortexing and a tenfold serial dilution was made to obtain 100 µg/ml, 10 µg/ml, 1 µg/ml, 0.1 µg/ml and 0.01 µg/ml stock solution of BBR or Lipo-BBR respectively. Ten µl of these BBR or Lipo-BBR solutions was added to 0.1 X 10^6^ cells such that BMMC were treated with either BBR alone (100 µg/ml, 10 µg/ml, 1 µg/ml, 0.1 µg/ml and 0.01 µg/ml) or Lipo-BBR (BBR lipoplexes) with 1.5, 0.15, 0.015, 0.0015 and 0.00015 µg of Lipofectamine 2000 respectively and incubated in a humidified CO_2_ incubator at 37 °C for 24 h.

BMMC (1 × 10^6^ or 5 × 10^6^ cells/ml) were seeded into round-bottomed 96 well plates in supplemented RPMI media. A 1 mg/ml solution of BBR was obtained by dissolving 1 mg of BBR in 1 ml PBS (BBR/PBS) or 1 ml 100% methanol (BBR/MeOH). A tenfold serial dilution was made to obtain 100 µg/ml, 10 µg/ml, 1 µg/ml, 0.1 µg/ml and 0.01 µg/ml BBR/PBS or BBR/MeOH. Ten µl of BBR/MeOH solutions of different concentrations or 100% methanol alone were added onto wells containing 0.1 × 10^6^ or 0.5 × 10^6^ cells, so that the final concentration of methanol was 10% in each of the wells. The plate was incubated in a humidified CO_2_ incubator at 37 °C for 24 h. Samples were prepared for flow cytometry analysis as described below.

All of the working stocks of inhibitors were made in PBS. BMMC (1 × 10^6^ cells/ml) were treated with Cytochalasin B (0.3 µM), 3-methyladenine (600 µM), Chloroquine (20 nM), Latrunculin B (3 µM) or an inhibitor cocktail of all the inhibitors for 1 h at 37 °C prior to treatment with 1 µg/ml BBR. Samples were collected at 24 h post treatment and were processed for flow cytometry as described below.

### IL-3 treatment

BMMC were cultured in supplemented RPMI (lacking IL-3) for 18 h. After 18 h, 0, 20 or 40 ng/ml mouse recombinant IL-3 (Peprotech, Rocky Hill, New Jersey, USA) was added followed by a simultaneous treatment with 10 µg/ml BBR for 3 or 24 h at 37 °C. After treatment, cells were processed for flow cytometry. For the proliferation assay, No. of cells/ml were estimated by utilizing a hemocytometer post trypan blue staining.

### Flow cytometry analysis of FcεRI and Kit expression and berberine internalization

BMMC were suspended in phosphate-buffered saline (PBS) supplemented with 0.5% bovine serum albumin (BSA) (PBS-BSA, Calbiochem Omnipur BSA fraction V) at 1.5 × 10^6^ cells/ml, and incubated with 0.006 µg/ml CD117 (c-Kit) PE (eBioscience, San Diego, California, USA) and 0.006 μg/ml FcεRIα APC (eBioscience) for 1 h at 4 °C. After washing with PBS-BSA twice, cells were re-suspended in 70 μl 0.5% BSA/0.05% sodium azide in PBS (PBS-BSA-sodium azide) and analyzed on a CytoFlex flow cytometer (Bechman Coulter, USA) by acquiring 20,000 events. Rat IgG2b κ PE (eBioscience) and Armenian Hamster IgG APC (eBioscience) were used as isotype controls.

To measure cell-associated BBR fluorescence, BMMC were washed twice with PBS-BSA and resuspended in 70 ul PBS-BSA-sodium azide and analyzed on a flow cytometer as described above. Fluorescence data was using a flow cytometer equipped with an Argon ion laser (488–514 nm) and bandpass filter to enable detection fluorescence emission at 516 nm (for BBR), 578 nm (for c-Kit PE) and 660 nm (for FcεRIα APC) by using the following parameters: FSC = 300 V and SSC = 300 V. 20,000 events per sample were acquired at a flow rate of 30 μl/minute at room temperature. As BMMC are very granular (high SSC) and large (high FSC) when compared to other immune cells, such as monocytes and lymphocytes, a well-defined cell population containing relatively high FSC and SSC were gated and analyzed as shown in Fig. 6C-i. This also serves to ensure that cell debris with low FSC and SSC values are eliminated from the analysis. Data was generated using FlowJo 10.6.2 software (Becton, Dickinson and Company, USA).

### Statistical analysis

Experiments were conducted at least in triplicate using three independent BMMC cultures started from the bone marrow of three animals and values represent mean of *n* = 3,4 or 5 ± standard error of the mean. *P* values were determined using Student t test and data was analyzed using GraphPad Prism software (https://www.graphpad.com/quickcalcs/ttest1.cfm).

## RESULTS

### Differentiated BMMC treated with berberine became fluorescent

Two of the most quintessential biomarkers of mast cells are the high affinity IgE receptor, FcεRI, which facilitates BMMC responses to antigens and the stem cell factor receptor, c-Kit (CD117), which is required for mast cell survival and differentiation. Mature mast cells are defined by the expression of these two surface receptors. Four-week-old BMMC cells were analyzed for the expression of surface biomarkers c-Kit and FcεRI receptors using flow cytometry (supplementary Fig. 1). It was found that 99% of the cells expressed these receptors showing that bone marrow progenitor cells had matured and differentiated into mast cells.

**Fig. 1:**
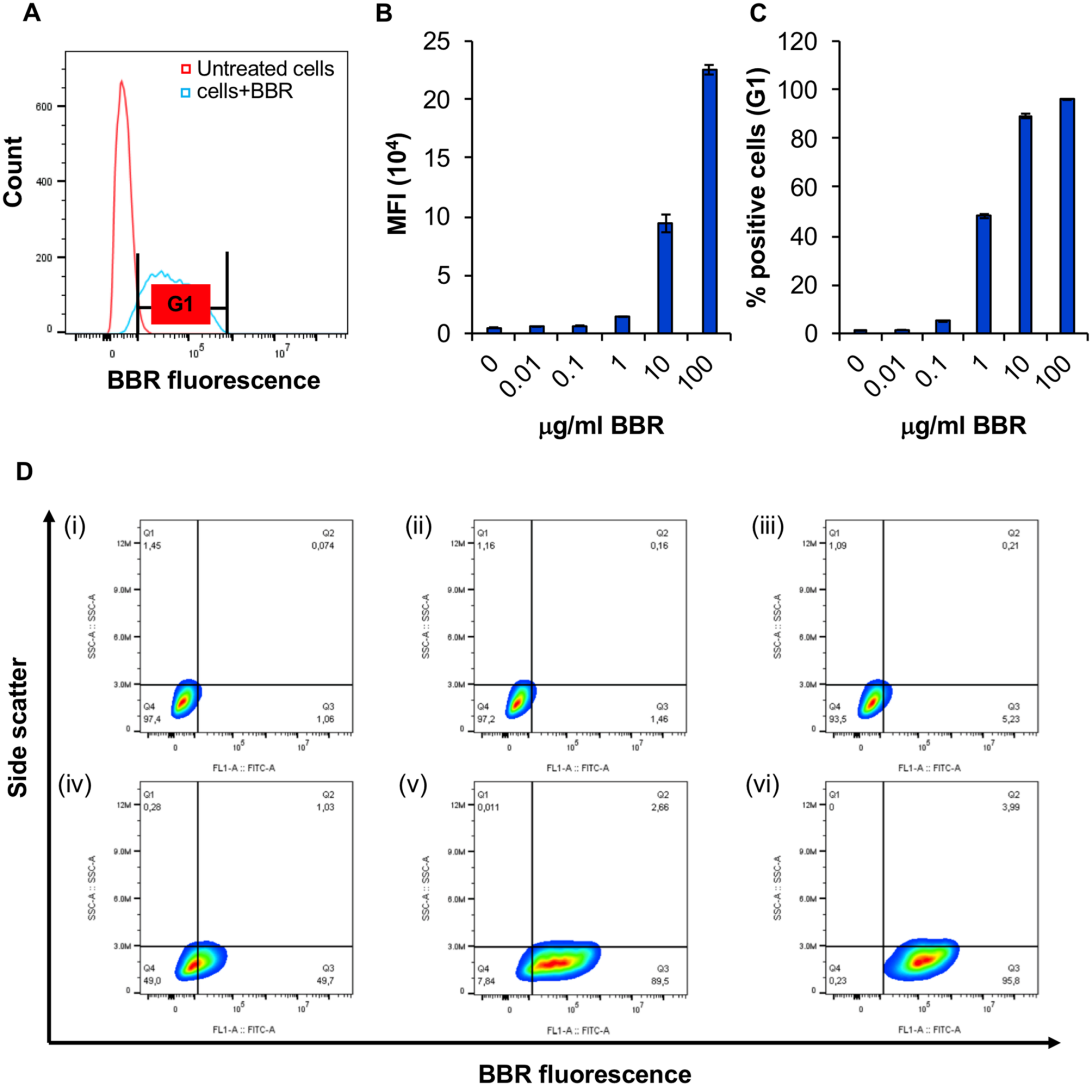
**Differentiated BMMC treated with berberine become fluorescent.** **A** Mean fluorescence intensity (MFI) following treatment (24 h) with BBR (0.1X 10^6^ cells with 10 μg/ml; *n* = 3). The red histogram represents untreated cells and the blue histogram represents cells treated with 10 μg/ml BBR. Gate “G1” was used to determine % positive cells compared to untreated cells. **B** Average MFI of cells that are fluorescent after treatment with different concentrations (0–100 μg/ml) of BBR for 24 h (*n* = 3). **C** Percent of fluorescent cells following treatment with BBR as in “B” (*n* = 3). **D** Fluorescent (X-axis) and side scatter (Y-axis) analysis of untreated (i) and cells treated with (ii) 0.01 μg/ml (iii) 0.1 μg/ml (iv) 1 μg/ml (v) 10 μg/ml and (vi) 100 μg/ml of BBR (24 h; *n* = 3)

When differentiated BMMC were exposed to 10 µg/ml BBR, there was an increase in BBR fluorescence as shown by a shift towards the right in the histogram overlay profile in Fig. 1A. In the subsequent experiments, fluorescence was found to increase in a dose-dependent manner (Fig. 1 B and C, *p* < 0.05) such that 100 µg/ml of BBR results in approximately 2.25 × 10^5^ mean fluorescence intensity (MFI, Fig. 1B) in almost 96 ± 0.06% of the cells (Fig. 1C). The dot plots in Fig. 1D, show that with increasing concentration of BBR a greater proportion of cells (from 1.46 to 95.8% cells) became fluorescent. Side scatter (SSC) and forward scatter (FSC) dot plot analysis, indicated that BMMC maintained their size and granularity up to 10 µg/ml (supplementary Fig. 2A). However, at a very high concentration of BBR (100 µg/ml), the FSC of the cells decreased, suggesting that the cells were not healthy after treatment with that concentration of BBR. To further look into the possibility if 100 µg/ml BBR is toxic to the BMMC, we conducted trypan blue staining and measured the % viability as well as the no. of live cells/ml following a 24 h treatment with 100 µg/ml BBR (supplementary Fig. 2B, C). Our results suggest that 100 µg/ml BBR had no significant effect on the % viability (supplementary Fig. 2B) as well as the number of live cells/ml (supplementary Fig. 2C).

**Fig. 2:**
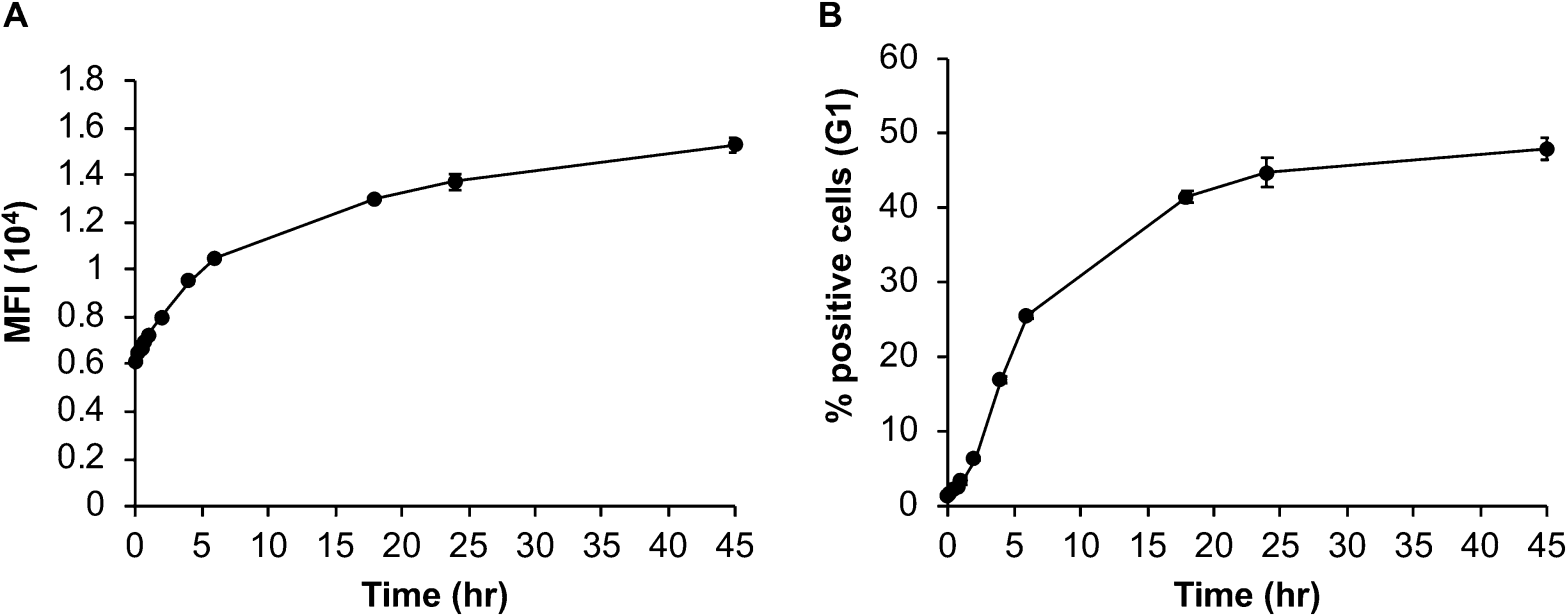
**BBR internalization increases over time.** **A** Average MFI of BMMC that were fluorescent after incubation with 1 μg/ml BBR for 0.25–45 h (*n* = 3) **B** Average Percent of fluorescent cells following treatment with BBR as in “A” (*n* = 3)

Next, we conducted a time course experiment (0–24 h) and found that BBR internalization increases over time as depicted by the increase in fluorescence over time (Fig. 2A). In addition, the percentage of cells incorporating BBR was also found to increase over time (Fig. 2B).

### BBR is internalized into BMMC rather than being adsorbed to their surface

**Fig. 3:**
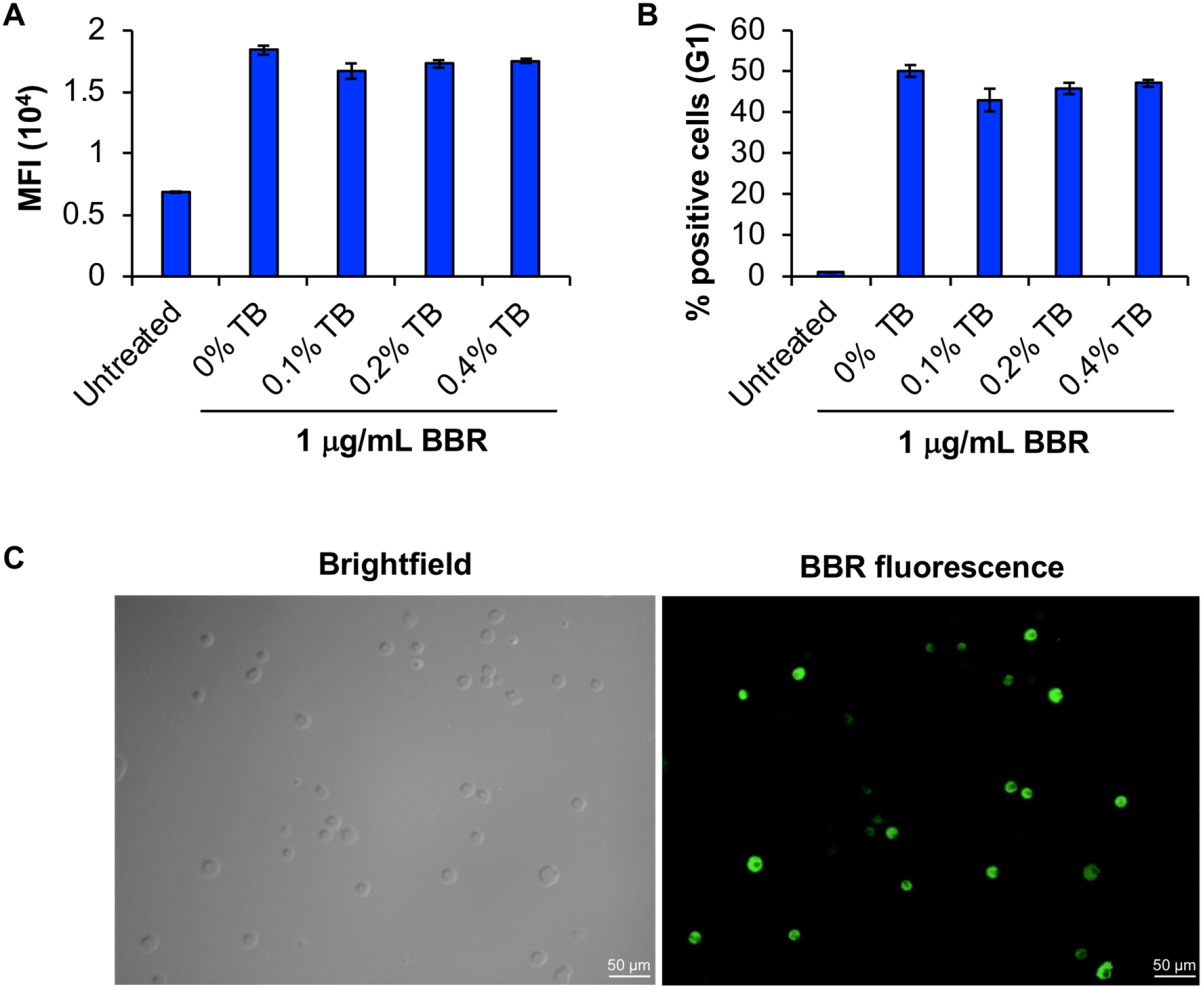
**BBR is internalized into BMMC rather than being adsorbed to their surface.** **A** Average MFI of cells that are fluorescent after incubation with 1 μg/ml BBR for 24 h (*n* = 3) and treated with 0%, 0.1%, 0.2% and 0.4% trypan blue (TB) prior to data acquisition. **B** Percent of fluorescent cells following treatment with BBR as in “A” (*n* = 3). (C) Fluorescence microscopic images of cells treated with 10 μg/ml BBR for 24 h and acquired at 12.6X magnification. Left panel represents the brightfield image and the right-hand side panel represents the same field view acquired using the FITC filter. Scale bar in each image represents 50 μm

To evaluate if BBR is internalized into BMMC, we conducted a trypan blue (TB) quenching assay. This assay is based upon the quenching of a closely associated fluorochrome via fluorescence resonance energy transfer from TB unless they are separated by a physical barrier [13]. Our results show that, TB was unable to quench the fluorescence emitted by BBR at any of the tested concentrations (0.1–0.4%), suggestive of the presence of a physical barrier such as the plasma membrane between the two. These results suggest that BBR is internalized into BMMC rather than being adsorbed to their surface. We further conducted fluorescence microscopic analysis and found that BBR fluorescence was associated with the interior of the cells (Fig. 3C), reinforcing the notion that BBR is internalized into BMMC.

### BBR endocytosis takes place in metabolically active cells

We further investigated if BBR internalization would be affected if cells are cultured in a metabolically inactive environment. For that BMMC were treated with BBR in supplemented RPMI media or PBS for 2 and 4 h. We found that, internalization efficiency of BBR (Fig. 3D) as well as the % BBR positive cells (Fig. 3E) was significantly reduced at both 2 and 4 h when cells were cultured in PBS versus the supplemented RPMI media. These results suggest that internalization of BBR takes place in a metabolically active environment.

**Fig. 4:**
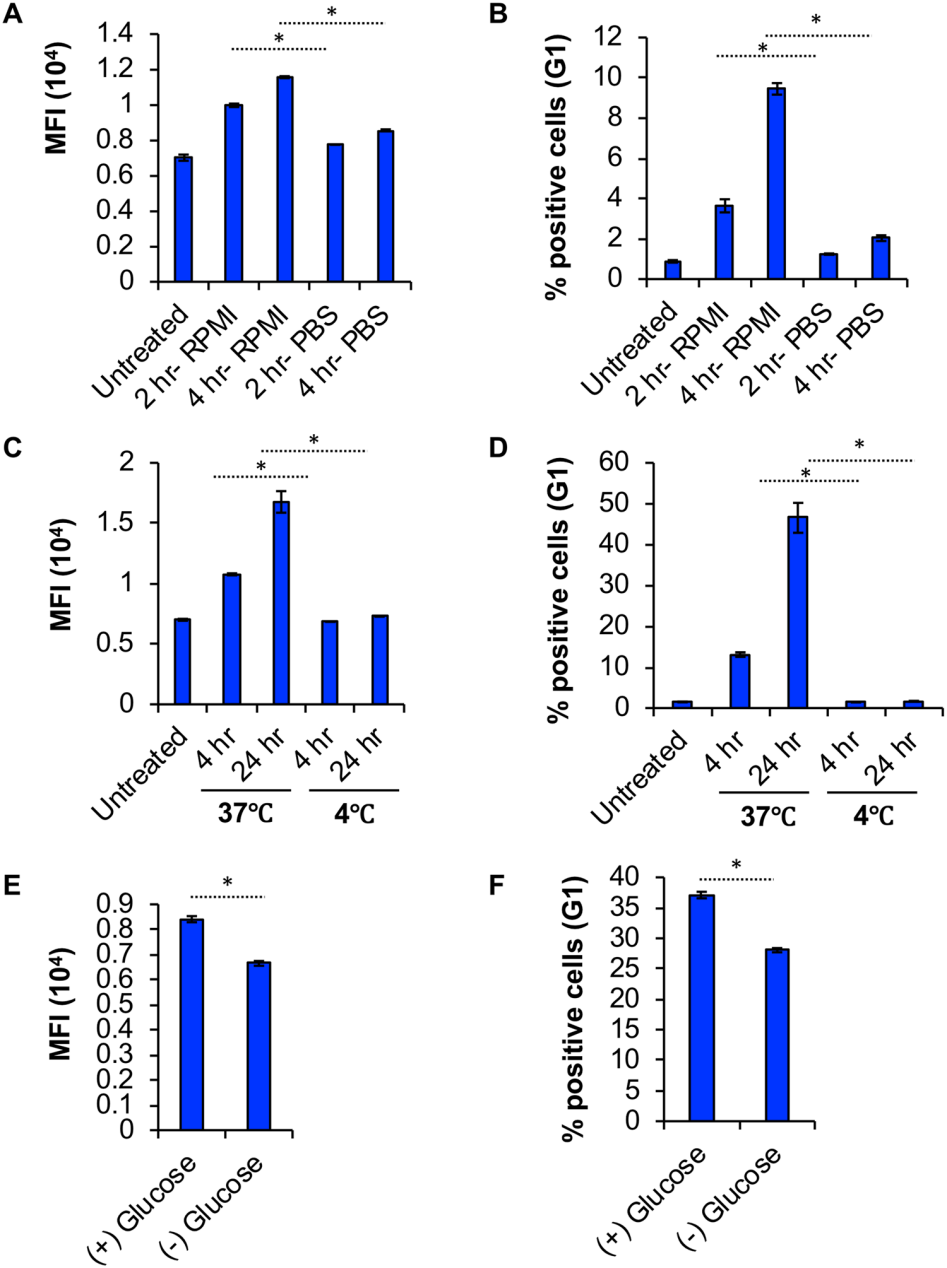
**BBR endocytosis takes place in metabolically active cells.** **A** Average MFI of cells treated with 1 μg/ml BBR for 2 h or 4 h in supplemented RPMI or PBS (*n* = 3). **B** % BBR positive cells treated as in “A”. **C** Average MFI of cells treated with 1 μg/ml BBR for 4 h or 24 h at either 4°C or 37°C (*n* = 3). **D** Percent of cells (G1) that are fluorescent after treatment with BBR (4 or 24 h) as in “C”. **E** Average MFI of cells that were either cultured in supplemented RPMI media [( +) Glucose)] or starved [(−) Glucose)] of Glucose for 20 h followed by treatment with 1 μg/ml BBR for 24 h. Background fluorescence was deducted from each treatment (*n* = 3). **F** Percent of cells (G1) that are fluorescent after treatment with BBR (24 h) as in “E”. Background fluorescence was deducted from each treatment. Statistically significant data with *p* < 0.05 is represented as an asterisk

We further wished to evaluate if BBR internalization is affected by temperature and glucose, factors that play essential role during energy-dependent endocytic processes. To investigate if temperature plays a role in BBR internalization into BMMC, we conducted the experiment at 4°C or 37°C and found that internalization efficiency was significantly reduced when cells were cultured at 4°C. There was minimal or no incorporation of BBR into BMMC at 4°C at both 4 h and 24 h post BBR treatment (Fig. 4A, B).

To look into the glucose dependency of BBR’s internalization process, cells were cultured in supplemented RPMI [( +) Glucose] or glucose free supplemented RPMI [(−) Glucose] media followed by treatment with BBR. At 24 h post BBR treatment, there was a significant reduction in BBR internalization. Altogether, these results suggest that BBR internalization into BMMC depends on temperature and that it requires glucose.

### Incorporation of BBR into lipoplexes inhibited internalization

**Fig. 5:**
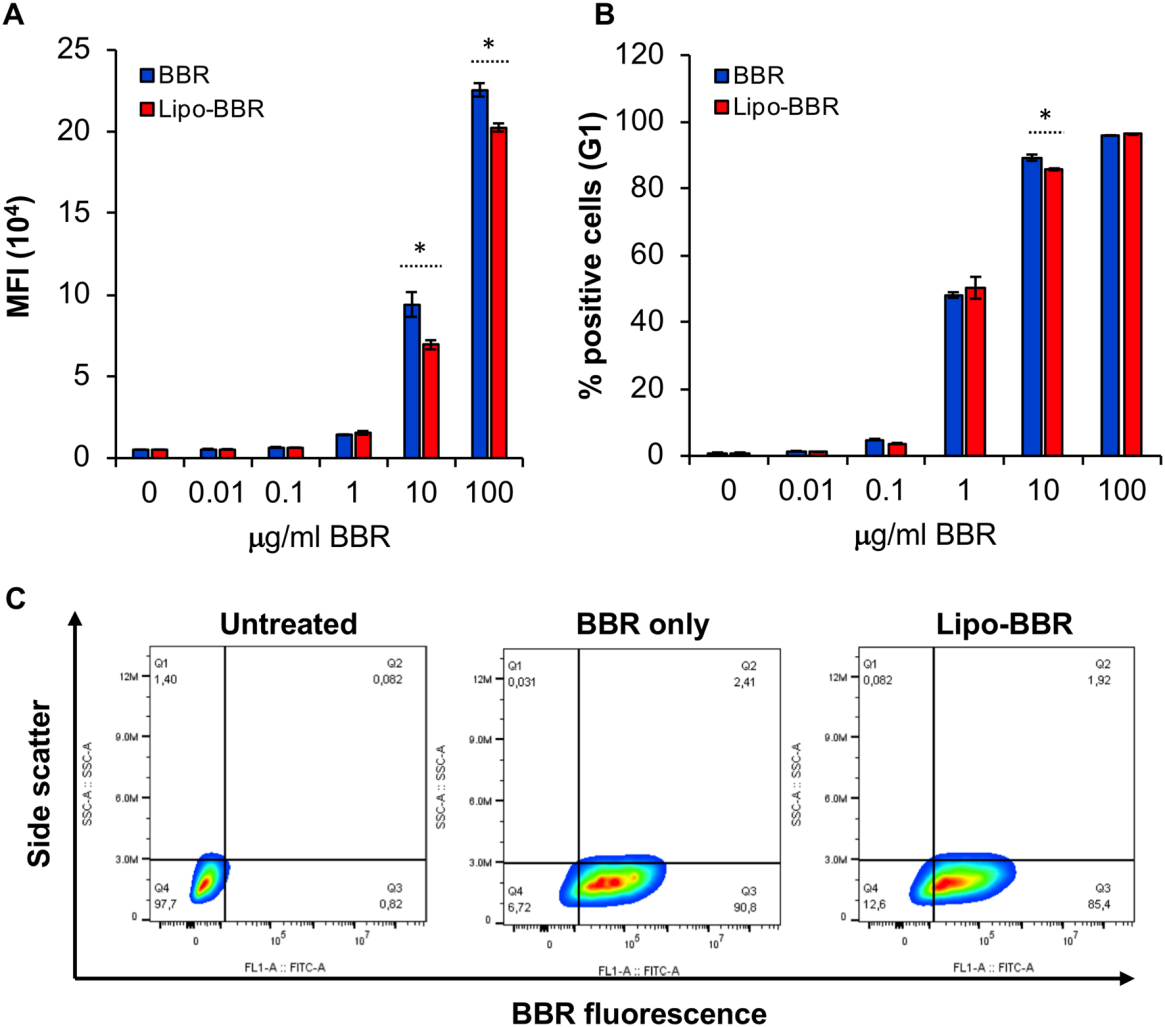
**Lipofectamine 2000 inhibits berberine internalization.** **A** Average MFI of cells that are fluorescent after treatment with different concentrations of BBR or Lipo-BBR for 24 h (*n* = 3). **B** Average % of cells (G1) that are fluorescent after treatment with different concentrations of BBR or Lipo-BBR for 24 h (*n* = 3). **C** Side scatter (Y-axis) and BBR fluorescence (X-axis) analysis of (i) untreated cells (i.e. cells treated with PBS) and cells treated with (ii) 10 μg/ml BBR alone (iii) or 10 μg/ml Lipo-BBR for 24 h (*n* = 3). Statistically significant data with *p* < 0.05 is represented as an asterisk

Lipid-based transfection reagents, such as Lipofectamine 2000 have been used to enhance the internalization of compounds and nucleic acids because they circumvent the clathrin-mediated endocytic pathways that deliver cargo to endolysosomes [14]. To determine if Lipofectamine 2000 could enhance internalization of BBR into mast cells, BMMC were incubated with BBR alone or with Lipofectamine 2000 (Lipo-BBR). Surprisingly, Lipofectamine 2000 inhibited BBR uptake. Increasing concentrations of Lipo-BBR facilitated internalization of BBR, but not to the extent when the cells were treated with BBR alone (Fig. 5A). There was a 26% and 10% reduction in the average MFI when cells were treated with Lipo-BBR compared to BBR alone (10 µg/ml and 100 µg/ml, respectively). This trend was also depicted by the % BBR positive cells (Fig. 5B). The dot plots in Fig. 5C show that incubation of BMMC with Lipo-BBR inhibits BBR internalization (compare percentage of positive cells in Q3 in panels ii and iii). When BMMC were incubated with 10 µg/ml BBR alone (panel ii), 90.8% of the cells were fluorescent whereas in the presence of 1.5 µg Lipofectamine 2000 (panel iii), BBR is incorporated in only about 85.4% of the cells. These results suggest that Lipofectamine 2000 blocks internalization of some BBR.

### BBR internalization is reduced when BMMC are fixed with methanol

**Fig. 6:**
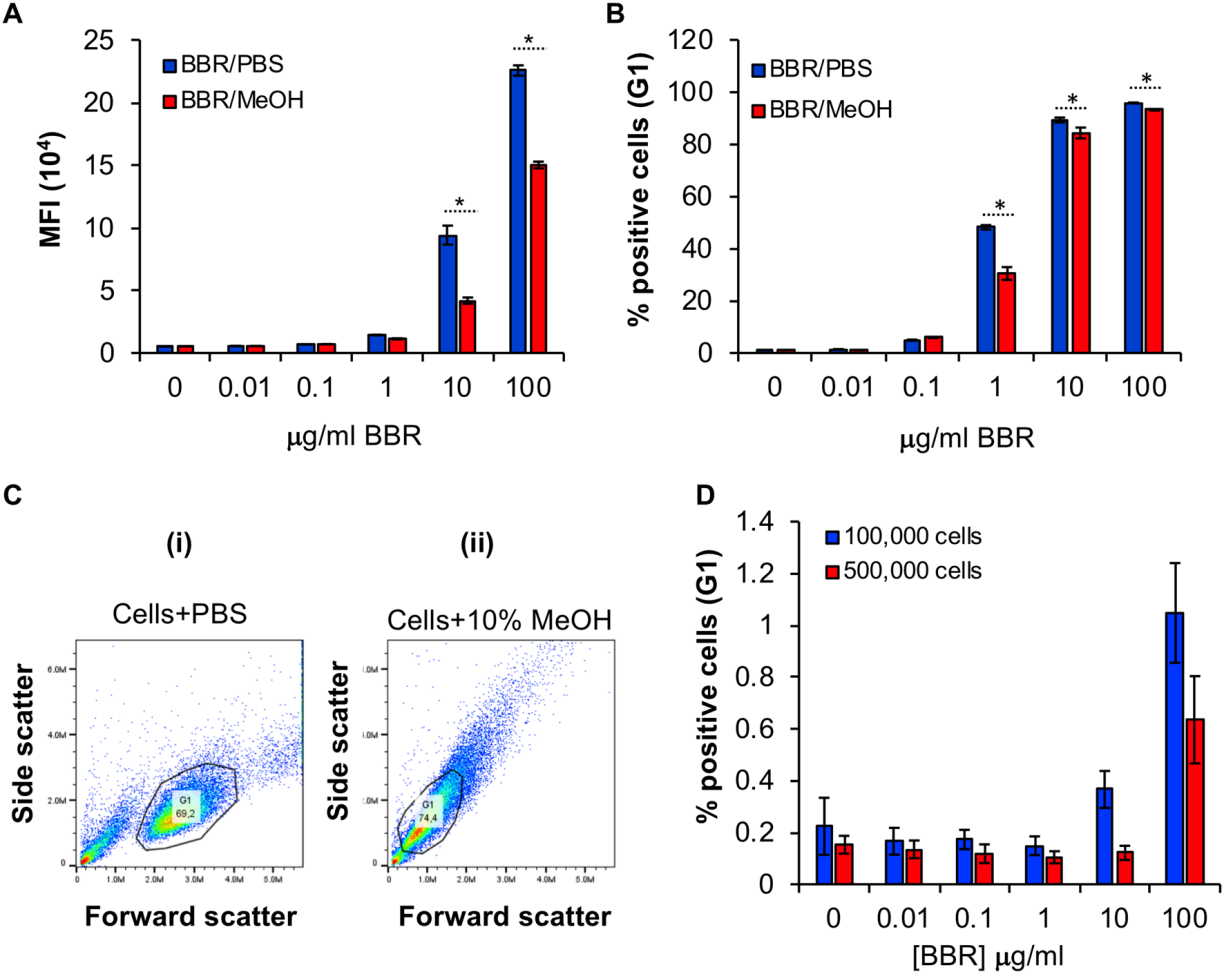
**BBR internalization is significantly reduced when BMMC are fixed with methanol. A** Average MFI of cells that are fluorescent after treatment with different concentrations of BBR/PBS or BBR/MeOH for 24 h (*n* = 3). **B** Average % of BBR positive cells after treatment as in “A”. **C** Forward scatter (X-axis) and side scatter (Y-axis) analysis of BMMC treated with PBS or 10% MeOH for 45 h. Note: the shift in the G1 population when cells are fixed with 10% methanol for 45 h. **D** Average % BBR positive cells (0.1 × 10^6^ or 0.5 × 10^6^) fixed with 10% methanol and treated with 0–100 μg/ml BBR for 45 h; *n* = 4. Statistically significant data with *p* < 0.05 is represented as an asterisk

To assess whether BBR was passively diffused into BMMC through Brownian motion, we fixed BMMC with methanol, to block all the active transport pathways and then treated with BBR to measure fluorescence of the BMMC. Since prolonged exposure to methanol can cause pore formation in cell membranes, we treated the cells with methanol and BBR simultaneously. We found that when cells were fixed with methanol, BBR internalization was significantly reduced by 56% and 33% by 10 and 100 µg/ml BBR treatment, respectively (Fig. 6A). Consistent with this observation, significantly fewer % of cells were found to be BBR positive (Fig. 6B) upon methanol fixation. By 45 h, we found that cells had become smaller and less dense as indicated by the dot plots in Fig. 6C (compare gated cell population G1 between panels i and ii). Interestingly, by 45 h BBR fluorescence was almost undetectable. Figure 6D shows that when cells are fixed with methanol for 45 h, there is undetectable or minimal incorporation of BBR into BMMC. These results suggest that BBR likely utilises an energy-dependent active transport mechanism to internalize into BMMC.

### BBR is internalized via endocytosis

**Fig. 7:**
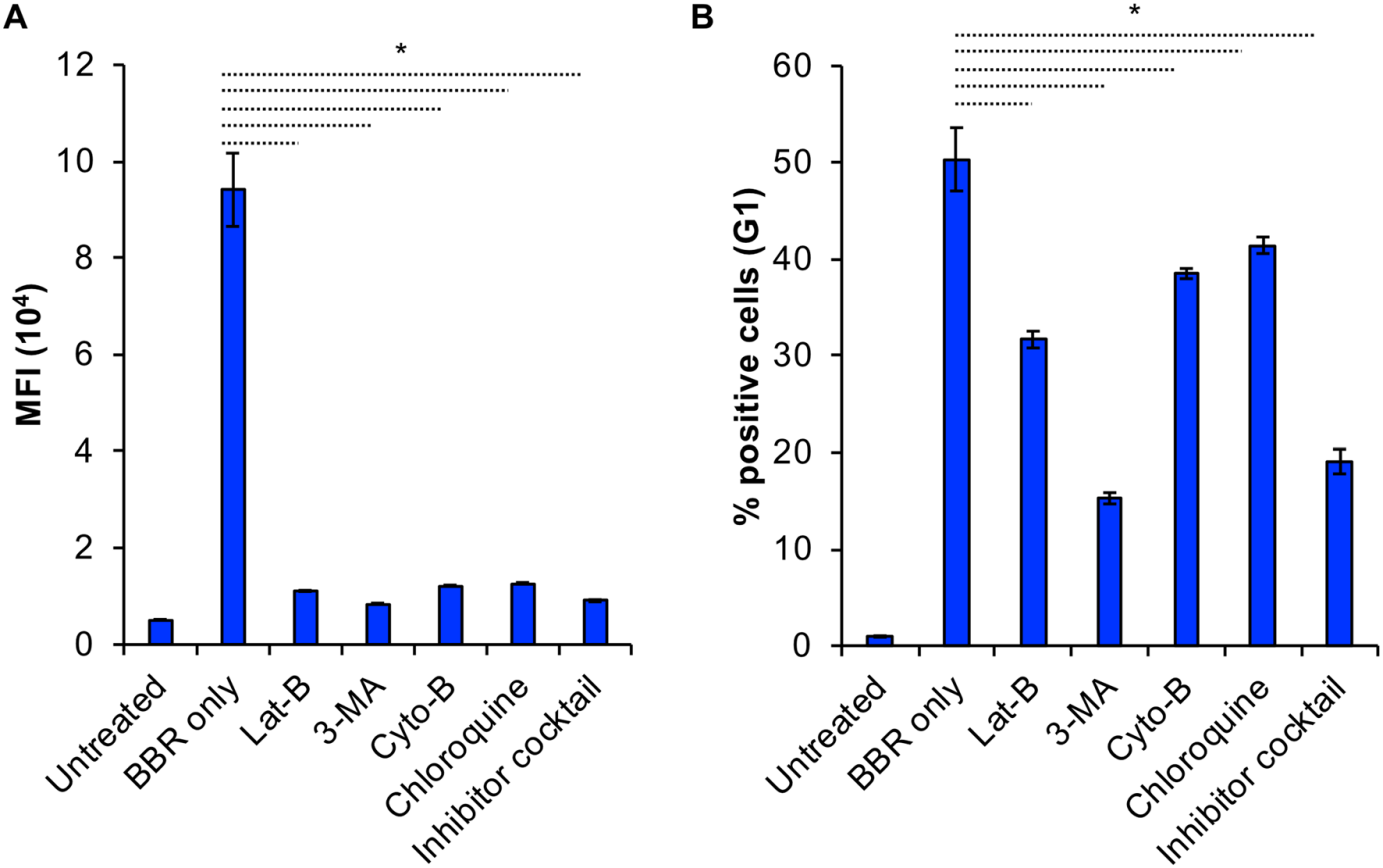
**BBR is internalized via endocytosis**. **A** BMMC were incubated with either PBS or 3 µM Latrunculin B (Lat B), 600 µM 3-methyladenine (3-MA), 0.3 µM Cytochalasin B (Cyto B),20 nM Chloroquine or a cocktail consisting of all the inhibitors dissolved in PBS for 1 h at 37°C. One hour post incubation BBR in PBS was added to a final concentration of 1 µg/ml followed by incubation at 37°C for 24 h. Average MFI of cells that are fluorescent after treatment (24 h) with either BBR alone or BBR in cells pre-incubated with inhibitors (*n* = 3). **B** Percentage of 0.1 × 10^6^ cells (G1) that are fluorescent after treatment (24 h) with either BBR alone or BBR in cells pre-incubated with inhibitors (*n* = 3). Statistically significant data with *p* < 0.05 is represented as an asterisk

Since BBR appeared to utilize an energy-dependent active transport pathway, BBR internalization by BMMC was analyzed following treatment with inhibitors of major endocytic and intracellular trafficking pathways. Our results show that BBR endocytosis was significantly reduced by Latrunculin B, Lat B (88%), cytochalasin B, Cyto B (87%), Chloroquine (86.5%) and 3-methyladenine, 3-MA ( (91%), indicating that actin polymerization, lysosomal pH and lysosomal self-degradation via the autophagy pathway was involved (Fig. 7A). Our results showed that, 3-methyladenine had the most significant effect in reducing BBR internalization. In contrast to 50 ± 3.3% cells being positive for BBR fluorescence when exposed to BBR alone, only about 15 ± 0.6% cells were fluorescent when BMMC was pre-incubated with 3-MA (Fig. 7B). Additionally, when cells were treated with a combination of all the inhibitors followed by BBR treatment, there was a 90% reduction in overall cell fluorescence (Fig. 7A, *p* < 0.05) as well as a substantial reduction in the percentage of positive cells (Fig. 7B, *p* < 0.05). These results suggest that BBR internalizes into BMMC by utilizing a mechanism that is associated with the autophagy-dependent endocytic pathway.

### BBR endocytosis is enhanced by IL-3 in a concentration-dependent manner

**Fig. 8:**
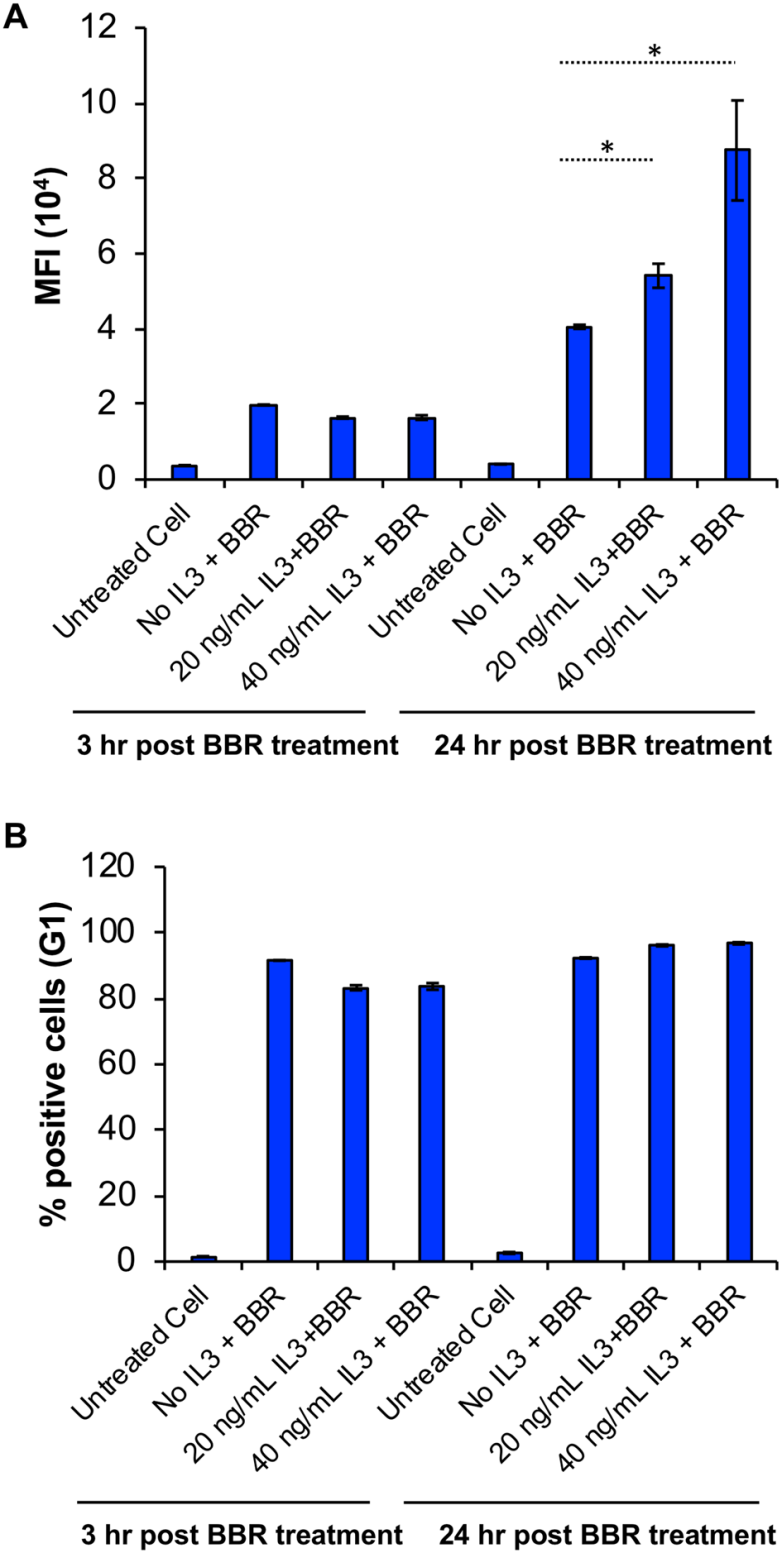
**BBR endocytosis is enhanced by IL-3 in a concentration-dependent manner**. **A** BMMC were cultured in IL-3 free supplemented RPMI media for 18 h. After 18 h, 0, 20 or 40 ng/ml IL-3 was added to the IL-3-deprived cells followed by incubation with 10 μg/ml BBR in PBS. Samples were collected at 3 h and 24 h post BBR treatment. Average MFI of cells that are fluorescent after treatment (3 h or 24 h) with BBR and 0, 20 or 40 ng/ml IL-3 (*n* = 3). **B** Graphical representation of percentage of 0.1 × 10^6^ cells (G1) that are fluorescent after treatment (3 h or 24 h) with BBR and 0, 20 or 40 ng/ml IL-3 (*n* = 3). Statistically significant data with *p* < 0.05 is represented as an asterisk

Given that IL-3 is a survival and differentiation factor for mouse mast cells [15], we evaluated the effects of IL-3 on BBR internalization. We found that IL-3 enhanced internalization of BBR into BMMC, such that cells treated with increasing concentrations of IL-3 for 24 h showed enhanced BBR uptake (Fig. 8A). However, since the percentage of cells that had internalized BBR did not change, neither with IL-3 exposure, nor with increasing concentrations of IL-3 (Fig. 8B), the data suggests that IL-3 increases the amount of BBR incorporation in a subpopulation of cells but does not increase the number of cells that are able to internalize BBR. Since IL-3 is a proliferative factor for BMMC, it is possible that IL-3 could stimulate quiescent BMMC into the proliferative stages of the cell cycle, thereby causing an increase in BBR internalization. To test this possibility, we measured BMMC proliferation after treatment with IL-3. Our results suggest that IL-3 did not cause an increase in BMMC proliferation compared to BMMC cultured in the absence of IL-3 (Supplementary Fig. 3).

## Discussion

Although our understanding of mast cell exocytosis is extensive, our conception of mast cell internalization and endocytic pathways, particularly for small molecules such as drugs and inhibitors, is poorly characterized. At a time when drug delivery applications are gaining significant momentum, it is essential that mast cell internalization of small molecules is better elucidated. In addition to being utilized as a cost-effective method for staining mast cell granules, BBR also has profound anti-inflammatory properties [16]. Despite its multifunctional roles, studies on BBR internalization by mast cells and intracellular trafficking is unknown. In this study, we examined the internalization mechanism of BBR by fully differentiated BMMC and characterized some of the endocytic pathways that this small alkaloid compound utilizes to internalize into mast cell intracellular compartments.

Endocytosis is an energy-dependent cellular process used for transport of extracellular cargo such as receptors, ligands, nutrients, hormones, and growth factors into the cytoplasm and can initiate downstream signalling to maintain cellular integrity and homeostasis [17]. Endocytosis begins on the cell membrane and is followed by different internalization pathways that are highly dependent on the actin cytoskeleton and intracellular vesicles [18–20]. The chemical and physical properties of the cargo initiate the vesicle formation and the extent of membrane deformity or invagination at the initial entry site determines the mechanism of endocytosis. Classically, endocytosis is either clathrin-mediated or clathrin-independent. While clathrin-mediated endocytosis involves the formation of clathrin coated endocytic vesicles around the cargo, there are several clathrin-independent endocytic mechanisms that involve protein complexes such as caveolae or rafts, mechanochemical GTPase dynamin, RhoA or Rac-1 GTPase or endophilin. Additionally, endocytic processes for uptake of large cargo such as phagocytosis or macropinocytosis involve internalization of large-sized particles or a large volume of the extracellular bulk phase, respectively [18–20]. Internalization of an endocytic vesicle is followed by the formation of early endosomes (EE), late endosomes (LE) and endolysosomes (EL). The EE ripen into LE. This is followed by further reduction in pH to create EL, where due to changes in the physiochemical properties, the encapsulated cargo is discharged.

In the present study, we found that BBR internalization was significantly reduced when BMMC were fixed in methanol (Fig. 6). This method of fixation extracts cholesterol from cell membranes and creates holes in the plasma membrane, and has been used to stain DNA with propidium iodide which freely diffuses through compromised cell membranes by utilizing an energy-independent passive transport pathway [21]. For this reason, we fixed our cells concurrently with exposure to BBR, thereby blocking the energy-dependent endocytic pathways while preserving the integrity of the plasma membrane. Methanol fixation blocked BBR internalization suggesting that an energy-dependent mechanism was involved. Further experiments using inhibitors of specific endocytic pathways showed that BBR is possibly internalized via actin-dependent endocytic pathways (Fig. 7), since Lat B and Cyto B partially blocked BBR internalization. Chloroquine, which interferes with endosomal pH, was also found to partially block BBR internalization suggesting that BBR internalization may rely on an endolysosomal (EL) pathway. Most interestingly, the autophagy inhibitor 3-MA was the most potent inhibitor of BBR internalization, suggesting that BBR internalization is dependent upon type III Phosphatidylinositol 3-kinases (PI3K). PI3K is a key regulator of intracellular vesicle trafficking and is critical for the endocytosis of some cell surface receptors such as epidermal growth factor, transferrin receptors and G protein-coupled receptors [22, 23]. PI3K is also important in pinocytosis [24], suggesting that BBR can also be internalized via pinocytosis. In mast cells, PI3K is necessary for exocytosis [25, 26] and shuttling of vesicles along the cytoskeletal network. However, endocytosis is regulated by Ca^2+^- binding proteins such as synaptotagmins (Syts) II, III, and IX as well as neuronal Ca^2+^ sensor 1 (NCS-1) [26]. Syts are localized along the endocytic network, where each homologue resides at a distinct endocytic organelle and controls a specific step in the endocytic network. Syt III resides on early endosomes (EE) and is required for the formation of endocytic recycling compartment (ERC), whereas, Syt II locates at an amine-free late endosomal/lysosomal compartment (LE/EL) and controls transportation from early to late endosomes. Both Syt IX and NCS-1 localize to the ERC and regulate the export of internalized cargo from the ERC towards the trans-Golgi network. Recycling through the ERC is necessary for secretory granule protein sorting as well as activation of MAPK and extracellular signal regulated kinases [26]. NCS-1 stimulates FcεRI- triggered exocytosis and release of arachidonic acid metabolites.

Current therapeutic strategies use lipid-based drug delivery technology where the payload (small molecular drug, DNA or RNA) is encased in a lipid matrix composed of one or more cationic lipids, thereby facilitating interaction with the negatively charged cell membrane. To be effective, these cationic liposomes must undergo cellular uptake, traffic through the intracellular vesicle machinery, escape the endosomal compartment and gain entry to the nucleus [27]. Over the past few years, a number of gene delivery systems based on polysaccharides, cationic lipids and cationic polymers such as poly(L lysine) or polyethylenimine [28] and multifunctional envelope-type nano-device [29] have been utilized as effective drug delivery systems. Most of these reagents highjack the microtubule network and use active, vesicle-mediated transport in a process used by some viruses [30]. However, this route often leads to the endolysosome (EL) where the liposomes are degraded along with their DNA payloads [31, 32]. Lipofectamine 2000, a commercially available lipofection reagent, composed of a 3:1 mixture of DOSPA (2,3‐dioleoyloxy‐*N*‐ [2(sperminecarboxamido)ethyl]‐*N*,*N*‐dimethyl‐1‐propaniminium trifluoroacetate) and DOPE (1,2-Dioleoyl-sn-glycero-3-phosphoethanolamine) [33], is able to efficiently avoid active intracellular transport along microtubules and the subsequent entrapment and degradation of the payload within acidic/digestive lysosomal compartments [14]. Lipofectamine is thought to move through the cell cytoplasm through random Brownian motion and gain access to the nucleus through a microtubule-independent mechanism. In fact, this process, contrary to some other formulations, can activate cellular stress responses [34] and heat shock protein promoters [35]. The hypothesis that BBR is internalized via energy-dependent, endolysosomal pathways is supported by our data showing that lipoplexes of Lipofectamine 2000 and BBR are not as readily internalized (Fig. 5). Since lipoplexes and Lipofectamine in particular are thought to circumvent the endocytic pathway and internalize their payload through Brownian diffusion across the plasma membrane [14], it is possible that this pathway is insufficient to deliver BBR to the necessary intracellular compartments. In fact, our data suggest that Lipofectamine 2000 is not an ideal reagent for facilitating internalization of drug cargos into BMMC perhaps due to non-ideal interactions between the lipid components of Lipofectamine 2000 and the unique phosphatidyl components of the mast cell membrane. In this regard, other drug delivery systems relying on cationic lipids, polysaccharides or cationic polymers such as poly(L lysine) or polyethylenimine and multifunctional envelope-type nano-devices may be a more effective drug delivery system.

One possible reason for the decreased BBR internalization in the presence of Lipofectamine could be that Lipofectamine decreases the metabolism of BMMC, thereby making them refractory to endocytosis. If so, this would reinforce our hypothesis that optimal BBR internalization requires metabolically active cells. Certainly, over 300 publications have shown at least a minor cytotoxic effect of Lipofectamine on various cell types, especially when used in higher concentrations and for longer periods of time [36–38]**.** The cytotoxic effect has been a major issue with these types of transfection reagents in many contexts and must be carefully controlled in experimental systems. Although our live/dead flow cytometric analysis did not indicate a large loss in BMMC viability following Lipofectamine treatment, it is possible that Lipofectamine could cause a small and undetectable reduction in BMMC metabolic activity that may interfere with BBR uptake.

Given the fact that IL-3 does not cause any significant changes in BMMC proliferation over a period of 24 h, we made an interesting observation in this study that IL-3 treatment of fully differentiated BMMC can enhanced BBR endocytosis. IL-3 is itself internalized when bound to the IL-3 receptor (IL-3R) via clathrin-dependent endocytosis [39]. Hence, it is possible that BBR uses this process as a Trojan horse to internalize into BMMC.

**Fig. 9:**
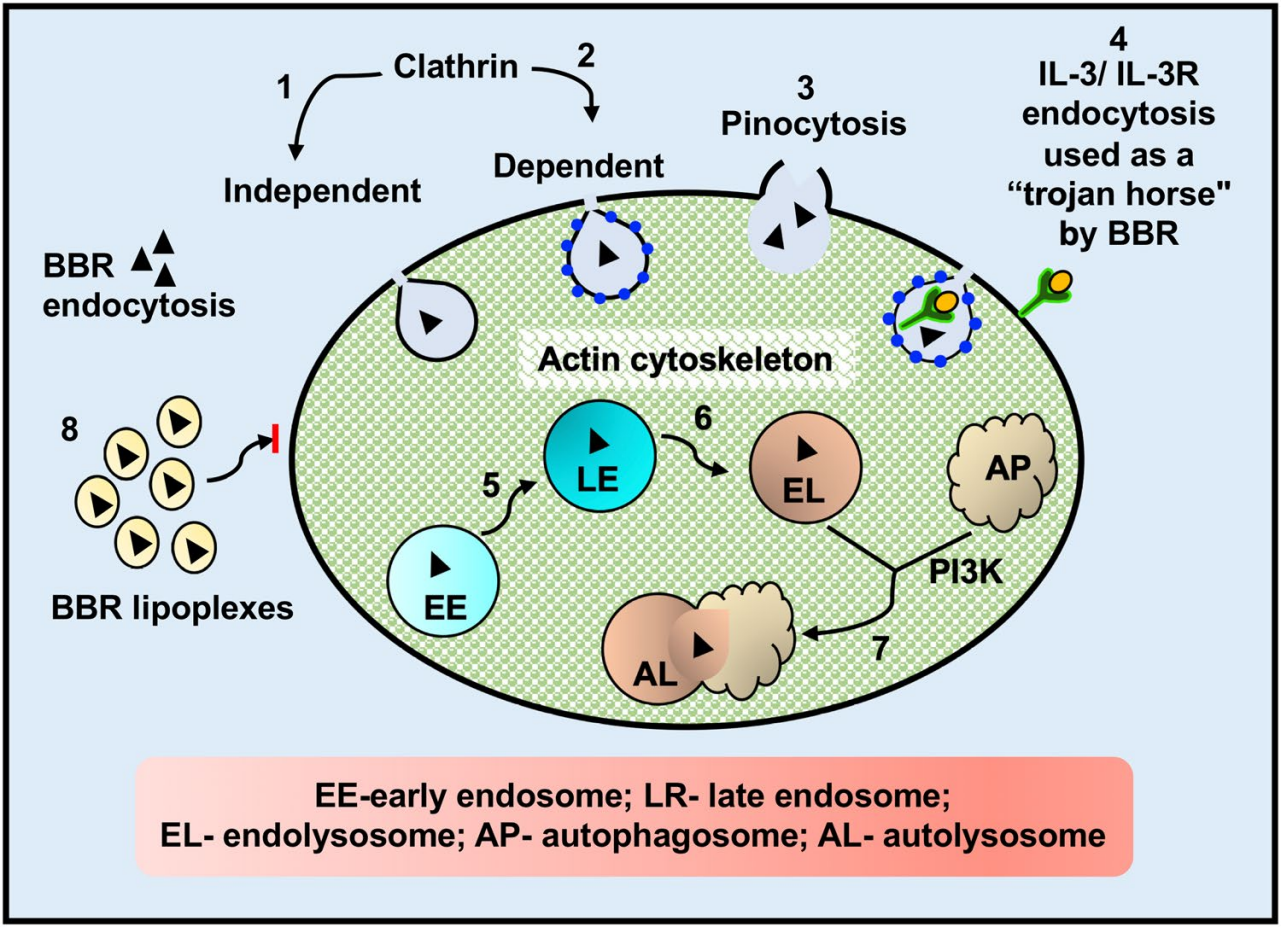
**Schematic representation showing possible energy-dependent internalization mechanisms of BBR into BMMC.** BBR could be internalized via clathrin-independent (1) or clathrin-mediated endocytosis (2) as well as pinocytosis (3) by utilizing the actin cytoskeleton shown in green. Berberine can also utilize clathrin-mediated endocytosis of IL-3R as a” trojan horse” to enter BMMC (4). Once internalized into early endosomes (EE) by utilizing a potential internalization pathway, the EE ripens into late endosomes (LE, 5) that ultimately matures to endolysosomes (EL) via further lowering of the pH (6). The EL and the autophagosomes (AP) fuse to form the autolysosomal compartment (AL), where PI3K plays an important role (7). It is to be noted that the vesicles move along the actin cytoskeletal network shown in green. BBR internalization is inhibited when it is incorporated into lipoplexes (8)

Altogether, our data suggest that internalization of BBR in resting and IL-3 activated BMMC may utilize energy-dependent endocytic pathways that may be dependent upon PI3K as outlined in Fig. 9. Our findings provide important new mechanistic insights into the internalization of benzylisoquinoline compounds by resting and activated mast cells and open new avenues for designing cell-targeted drug delivery technologies and therapeutics.

## Supplementary Information

Below is the link to the electronic supplementary material.

Supplementary file1 (TIFF 32874 KB)

Supplementary file2 (TIFF 32874 KB)

Supplementary file3 (TIFF 32874 KB)

Supplementary file4 (DOCX 13 KB)

## Abbreviations

BBR: Berberine chloride
Lipo: Lipofectamine 2000
BMMC: Bone marrow derived mast cell
IL-3: Interleukin-3
Lat B: Latrunculin B
3-MA: 3-methyladenine
Cyto B: Cytochalasin B
MFI: Mean fluorescence intensity
SSC: Side scatter
FSC: Forward scatter
PI3K: Phosphatidylinositol 3-kinases
