# Supplementary figures and images for: Internalization of benzylisoquinoline alkaloids by resting and activated bone marrow-derived mast cells utilizes energy-dependent mechanisms
Source: Inflamm Res. 2022 Jan 25;71(3):343–56. doi: 10.1007/s00011-021-01526-2 (PMC8897387; doi:10.1007/s00011-021-01526-2)

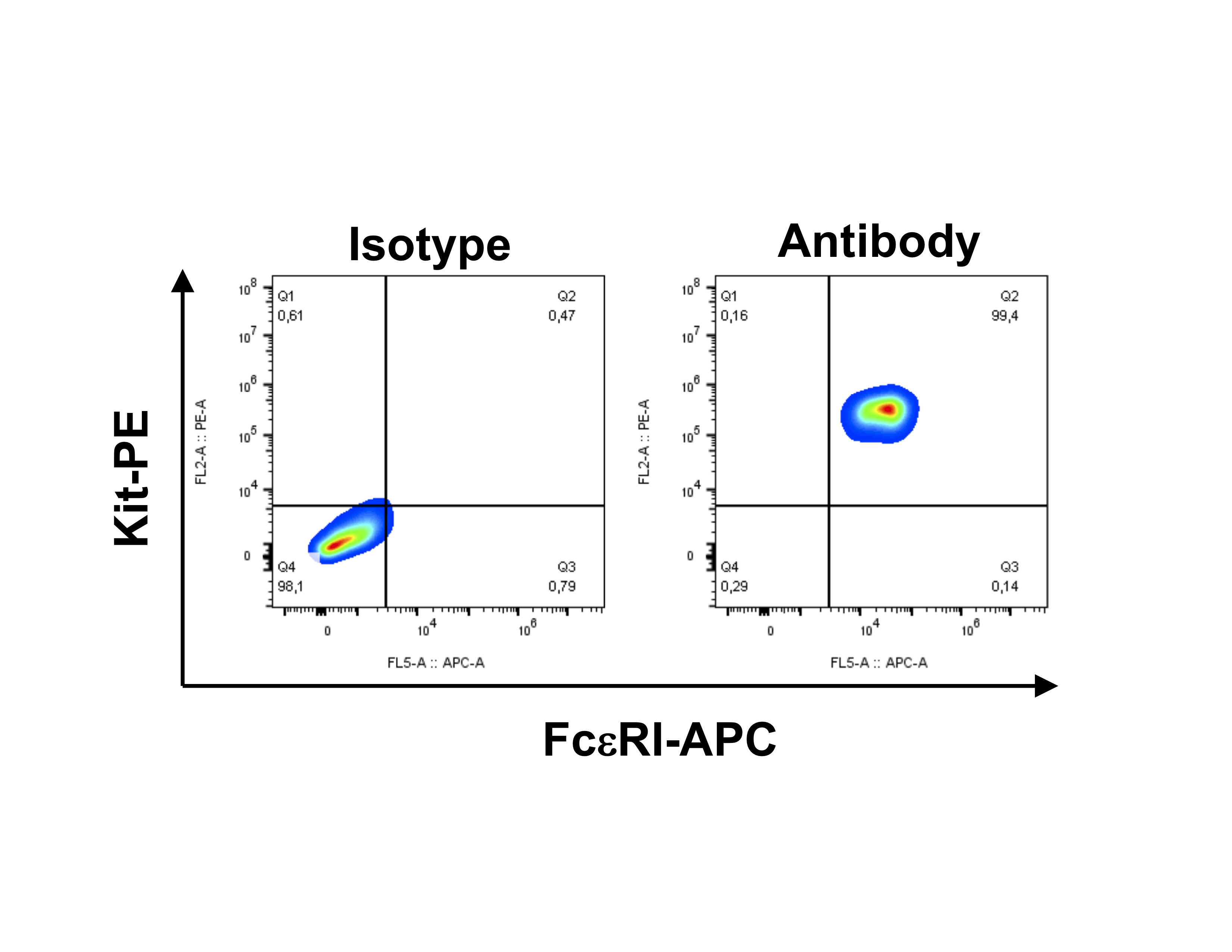

Supplement: Supplementary file 1 — Supplementary file1 (TIFF 32874 KB) [file 11_2021_1526_MOESM1_ESM.tiff]

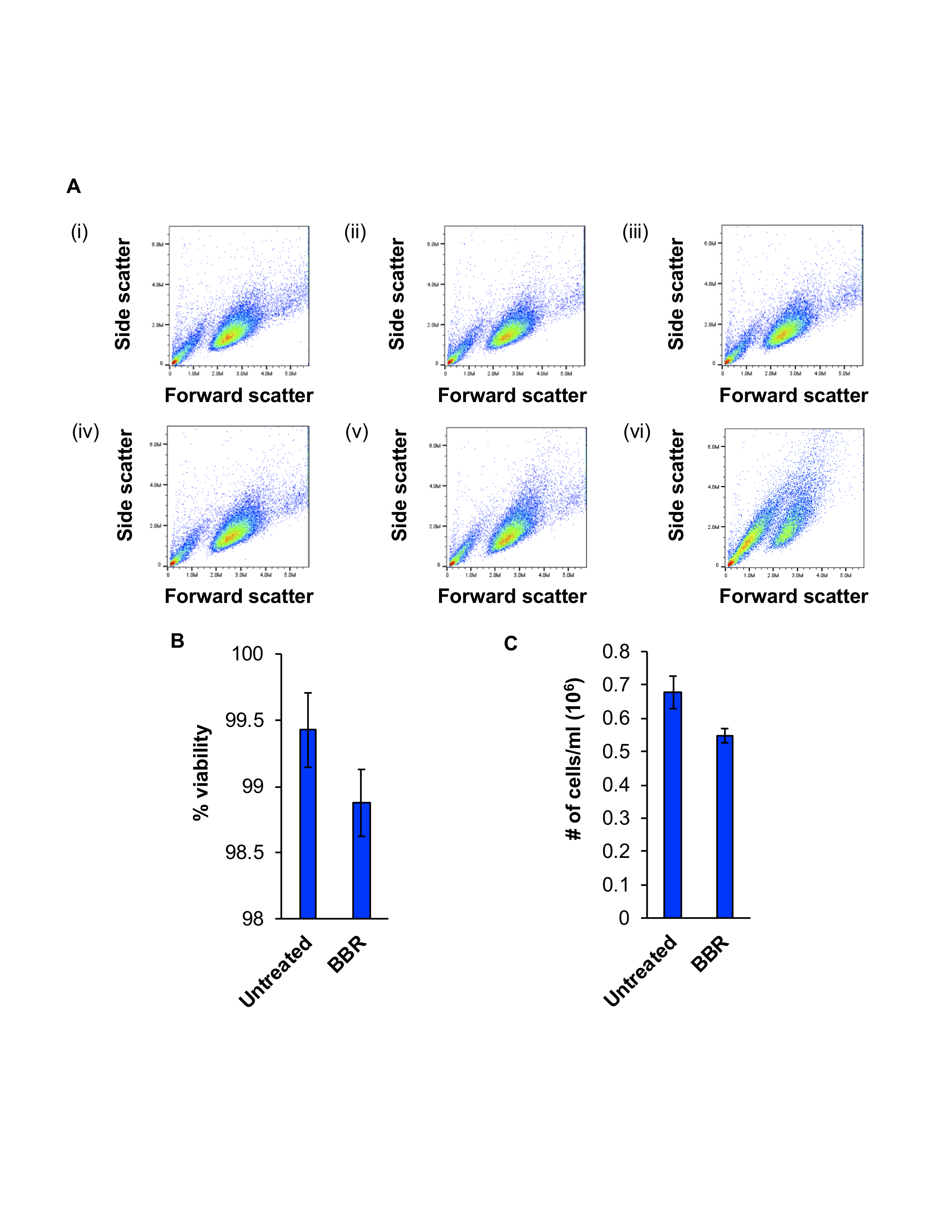

Supplement: Supplementary file 2 — Supplementary file2 (TIFF 32874 KB) [file 11_2021_1526_MOESM2_ESM.tiff]

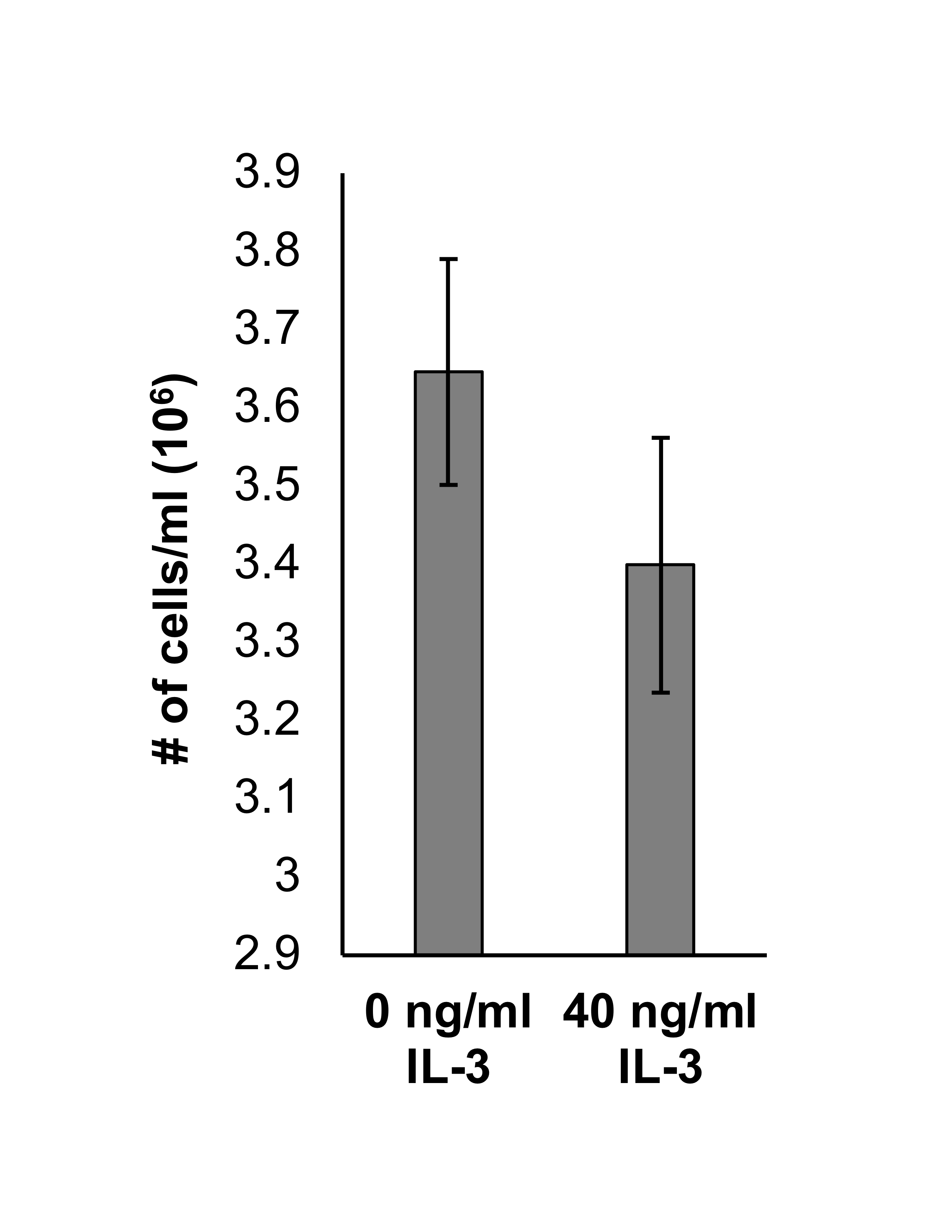

Supplement: Supplementary file 3 — Supplementary file3 (TIFF 32874 KB) [file 11_2021_1526_MOESM3_ESM.tiff]
