# Supplementary material for: Internalization of benzylisoquinoline alkaloids by resting and activated bone marrow-derived mast cells utilizes energy-dependent mechanisms
Source: Inflamm Res. 2022 Jan 25;71(3):343–56. doi: 10.1007/s00011-021-01526-2 (PMC8897387; doi:10.1007/s00011-021-01526-2)
Supplement: Supplementary file 4 — Supplementary file4 (DOCX 13 KB) [file 11_2021_1526_MOESM4_ESM.docx]

**Legends to Supplementary Figures**

**Supplementary Figure 1**: **Expression of FcεRI and Kit in fully differentiated 4 weeks-old resting BMMC by flow cytometry.**

Kit-PE (Y-axis) versus FcεRI-APC (X-axis) dot plot of 0.1X 10^6^ BMMC stained with 0.006 µg/ml of each of the isotype controls or antibodies.

**Supplementary Figure 2. Effects of BBR on BMMC viability**

(A) Side scatter (Y-axis) and forward scatter (X-axis) analysis of 0.1X 10^6^ BMMC treated with PBS (i) or 0.01 (ii), 0.1 (iii), 1 (iv), 10 (v), 100 (vi) μg/ml BBR dissolved in PBS for 24 hr (n=3). (B) 100,000 BMMC were treated with 100 μg/ml BBR for 24 hr followed by trypan blue staining to measure (B) % viability and (C) No. of live cells/ml (n=3).

**Supplementary Figure 3**. **IL-3 proliferation assay.**

0.1X 10^6^ BMMC were cultured in IL-3 free supplemented RPMI media for 18 hr, followed by treatment with wither 0 or 40 ng/ml IL-3. 24 hr post treatment, cells were stained with trypan blue and proliferation was estimated by evaluating the no. of cells/ml by using a haemocytometer (n=3).
